# (1*E*,3*E*)-1,4-Dinitro-1,3-butadiene—Synthesis, Spectral Characteristics and Computational Study Based on MEDT, ADME and PASS Simulation

**DOI:** 10.3390/molecules29020542

**Published:** 2024-01-22

**Authors:** Mikołaj Sadowski, Beata Synkiewicz-Musialska, Karolina Kula

**Affiliations:** 1 Department of Organic Chemistry and Technology, Cracow University of Technology, Warszawska 24, 31-155 Cracow, Poland; mikolaj.sadowski@doktorant.pk.edu.pl; 2 Łukasiewicz Research Network–Institute of Microelectronics and Photonics, Zabłocie 39, 30-701 Cracow, Poland; beata.synkiewicz.musialska@imif.lukasiewicz.gov.pl

**Keywords:** nitrodiene, synthesis, spectral characteristics, DFT reactivity indices, ADME, PASS

## Abstract

The chemistry of conjugated nitrodienes is becoming increasingly popular. These molecules are successfully applied in cycloaddition to synthesize six-membered rings in Diels-Alder reactions. Nitrodienes can be also applied to obtain bis-compounds in [3+2] cycloaddition. Moreover, the presence of a nitro group in the structure provides a possibility of further modification of the products. The simplest symmetrical representative of conjugated nitrodienes is (1*E*,3*E*)-1,4-dinitro-1,3-butadiene. Although the first mentions of the compound date back to the early 1950s, the compound has not yet been examined thoroughly enough. Therefore, in this article, a comprehensive study of (1*E*,3*E*)-1,4-dinitro-1,3-butadiene has been described. For this purpose, an experimental study including the synthesis process as well as an evaluation of the spectral characteristics has been conducted. So as to better understand the properties of this compound, a computational study of reactivity indices based on MEDT and also an assessment of pharmacokinetics and biological activity according to ADME and PASS methodologies have been made. On this basis, some future application trends of (1*E*,3*E*)-1,4-dinitro-1,3-butadiene have been proposed.

## 1. Introduction

The chemistry of nitro group-containing compounds has been of great interest to scientists for many years [1]. These kinds of structures are extremely attractive building blocks in modern organic synthesis. It is due to the presence of a nitro group in the molecule which creates many possibilities for its transformation. Thanks to this, it is possible to synthesize many nitrogen-containing connections like amines, hydroxylamines, oximes, nitriles, diazocompounds and others as well as to obtain carbonyl compounds [2]. Among all nitro compounds, a class of conjugated nitroalkenes (CNAs) deserves special attention [3,4]. These compounds exhibit various biological activities such as antibacterial [5,6] or antifungal [7]. This makes CNAs often used in many reactions such as Michael addition [8,9] as well as cycloaddition (CA) reactions, to synthesize heterocycles, especially for the preparation of five-membered rings [10] like pyrazolines [11,12], isoxazolines [13,14,15,16] or pyrrolidine [17,18], and six-membered rings in the Diels-Alder reaction [19,20,21].

In recent times, another class of nitro-unsaturated organic compounds—conjugated nitro dienes (CNDs)—are increasing in popularity [22,23]. Due to their structure, these compounds offer many more possibilities for transformation compared to CNAs [24].

**Scheme 1:**
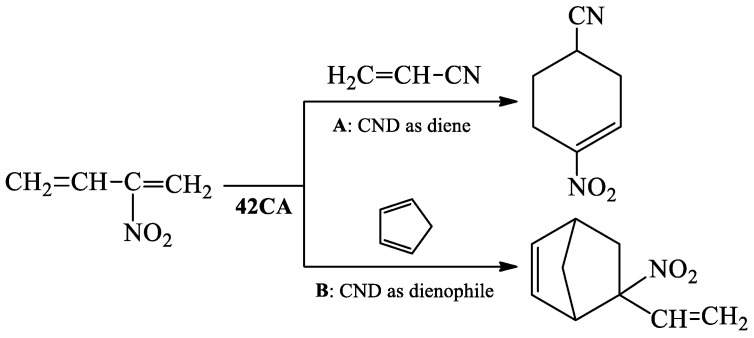
Application of a conjugated nitrodiene (CND) in Diels-Alder reactions in a double role.

Undoubtedly, CNDs are a primary component in the Diels-Alder ([4+2] cycloaddition, 42CA) reactions [25]. Most often, these compounds play a role of a standard diene (Scheme 1, pathway A). However, there are known cases of reactions where CNDs are applied in Diels-Alder reactions as dienophiles (Scheme 1, pathway B). 

**Scheme 2:**
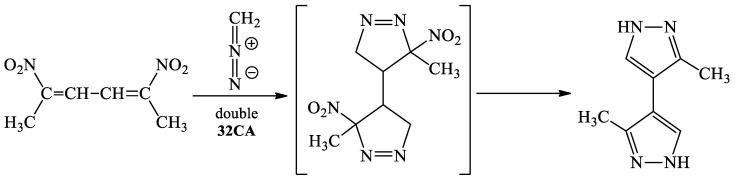
Application of a conjugated nitrodiene (CND) in [3+2] cycloaddition reaction to obtain a symmetric biheterocyclic system.

What is more, CNDs can be also applied in reactions of [3+2] cycloaddition (32CA) with various three-atom components (TACs) [26]. As a consequence, it is possible to obtain symmetric biheterocycles (Scheme 2) which may successfully be used in medicine [27] as well as in optoelectronics [28] as highly efficient photovoltaic materials [29] or solar cells [30] and also a useful organic ingredient improving the structural, thermal and dielectric properties in glass-ceramic composites for the dielectric industry in Low Temperature Cofired Ceramic (LTCC) Technology [31,32,33]. 

**Scheme 3:**
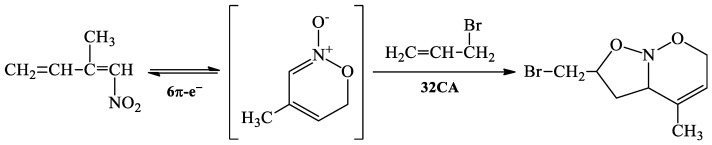
Application of a conjugated nitrodiene CND 6π-electrocyclization process to obtain a cyclic nitronate.

Examples of heterocycles obtained via the processes of 6π-electrocyclization (6π-e^−^) of CNDs are also found in the literature [22]. This protocol of ring closure can be useful to synthesize cyclic nitronates, which may react with ethylene systems creating final products (Scheme 3) [34].

However, the number of examples of the use of CNDs in CA reactions as well as in 6π-e^−^ processes is strongly limited. Reactions described in the literature that tackle the application of these compounds in organic synthesis are predominantly limited to nucleophilic additions and substitutions with thiols and amines; there are fewer examples of electrophilic additions of halogens [22,23,24,25]. Therefore, the use of CNDs as building blocks to obtain heterocyclic systems is an attractive course of research and determines one of the most promising trends in current organic synthesis. 

Despite the fact that the chemistry of CNDs has been known since the middle of the twentieth century, there are still many gaps in the synthesis and spectral characteristics of these compounds [23]. What is more, numerous protocols and reports presented in the scientific literature are incomplete. 

**Figure 1:**
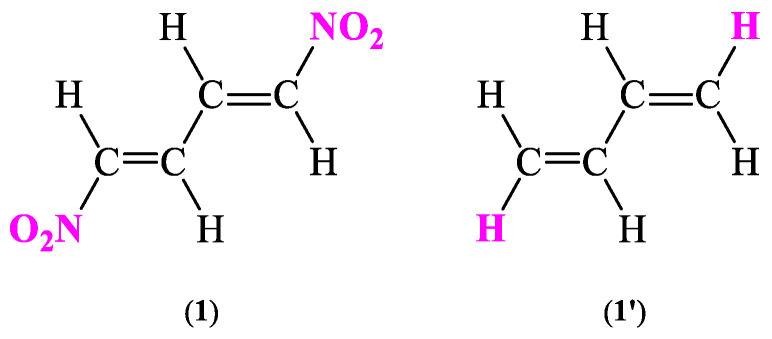
The structures of tested (1*E*,3*E*)-1,4-dinitro-1,3-butadiene (**1**) and *S*-*trans*-1,3-butadiene (**1′**) as the simplest analogues of nitrodiene **1**.

Seeing the wide applicational potential of CNDs in a modern heterocyclic chemistry, we decided to carry out a comprehensive study of one compound from the class. (1*E*,3*E*)-1,4-dinitro-1,3-butadiene (**1**) was selected as the research object (Figure 1). Compound **1** was chosen for several reasons. Firstly, nitrodiene **1** is representative of the simplest symmetrical CND systems. The compound **1** was successfully tested in several reactions, mostly in nucleophilic addition and nucleophilic substitution [23,25]. However, two examples of the application of (1*E*,3*E*)-1,4-dinitro-1,3-butadiene (**1**) in Diels-Alder reactions are available in the literature [26]. What is more, there are reports that compound **1** is one of the few nitrodienes that has biological properties. In 1970, Durden et al. [35] described that (1*E*,3*E*)-1,4-dinitro-1,3-butadiene (**1**) can be active against *Staphylococcus Aureus* and *Aspergillus Niger*. In the same research, the authors reported that nitrodiene **1** was tested against *Uromyces Phaseoli*. According to in vivo tests, compound **1** is a promising candidate to be applied in agrochemical industry as a means to prevent bean rust [35]. In view of the above, systematizing information and filling information gaps regarding the (1*E*,3*E*)-1,4-dinitro-1,3-butadiene (**1**) is justified.

As a part of the research, a comprehensive experimental-computational study was carried out. For this purpose, (1*E*,3*E*)-1,4-dinitro-1,3-butadiene (**1**) was synthesized. During the process, necessary modifications of the synthesis protocol were introduced to increase the efficiency of obtaining nitrodiene **1**. Then, the spectral analyses were performed to confirm the structure of the obtained system. Research of the geometric isomerism aspects was also carried out. In turn, in the computational part, the reactivity of title compound **1** was thoroughly studied. For this purpose, selected aspects of Molecular Electron Density Theory (MEDT) [36] were used. The influence of the presence of the nitro group on the reactivity properties was also examined by comparing nitrodiene **1** with its analogue, the simplest representative of dienes, which is *S*-*trans*-1,3-butadiene (**1′**) (Figure 1). Finally, in order to address the biological potential of (1*E*,3*E*)-1,4-dinitro-1,3-butadiene (**1**), analyses of physicochemical descriptors, pharmacokinetic properties, drug-like nature and medicinal chemistry friendliness, based on selected aspects of ADME (Absorption, Distribution, Metabolism, Excretion) and PASS (Prediction of Activity Spectra for Substances) were performed. 

## 2. Results and Discussion

### 2.1. Synthetic and Computational Aspects of (1E,3E)-1,4-Dinitro-1,3-butadiene Preparation 

#### 2.1.1. Synthesis Protocol and Its Necessary Modifications

**Scheme 4:**
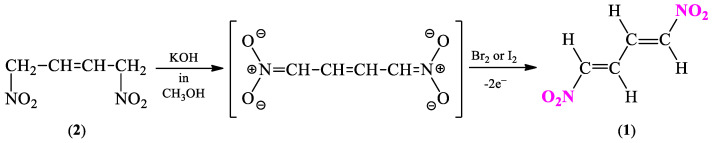
Synthesis of (1*E*,3*E*)-1,4-dinitro-1,3-butadiene (**1**) via electrochemical processes.

Generally, in the literature, two synthesis methods of (1*E*,3*E*)-1,4-dinitro-1,3-butadiene (**1**) are available [23,25]. One of them includes an electrochemical process of direct oxidation of 1,4-dinitrobut-2-ene (**2**) (Scheme 4) by bromine or iodine in the presence of a methanolic solution of KOH [37].

**Scheme 5:**
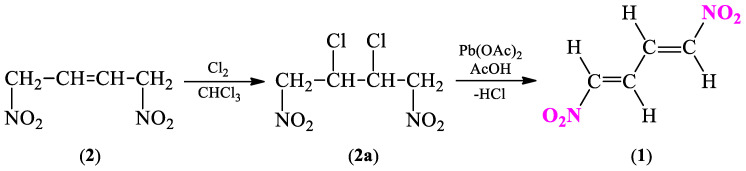
Synthesis of (1*E*,3*E*)-1,4-dinitro-1,3-butadiene (**1**) via dehydrochlorination reaction of 2,3-dichloro-1,4-dinitrobutane (**2a**).

Definitely, the most popular alternative methods are the thermal eliminations of different fragments like hydrogen halides or acetic acid from 1,4-dinitrobutane analogues [23,25]. The reaction can be carried out by two different paths. The first starts from 1,4-dinitrobut-2-ene (**2**), similarly to previous example (Scheme 5). In this case, dinitroalkene **2** is chlorinated with gaseous chlorine, in the presence of iodine to obtain 2,3-dichloro-1,4-dinitrobutane **2a**. In the next stage, the process of dehydrochlorination of compound **2a** in a presence of lead(II) acetate in glacial acetic acid is carried out [38].

**Scheme 6:**
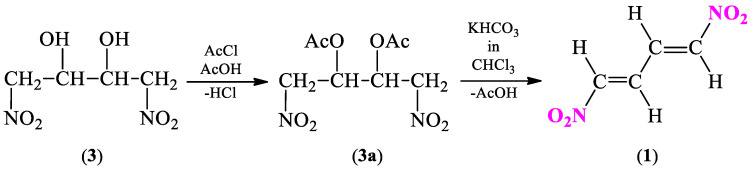
Synthesis of (1*E*,3*E*)-1,4-dinitro-1,3-butadiene (**1**) via dehydro-acetylation reaction of 2,3-diacetoxy-1,4-dinitrobutane (**3a**).

Another alternative based on an elimination reaction requires the creation of a conjugated system based on 1,4-dinitrobutane-2,3-diol (**3**) (Scheme 6). The first step includes an acylation process of diol **3** to 2,3-diacetoxy-1,4-dinitrobutane (**3a**). In the next stage, an elimination of two acetic acid molecules from ester **3a** in the presence of potassium bicarbonate in chloroform is carried out [35,39,40,41].

**Scheme 7:**
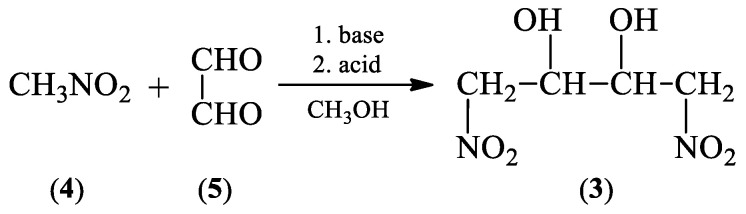
Synthesis of 1,4-dinitrobutane-2,3-diol (**3**) via a condensation reaction between nitromethane (**4**) and glyoxal (**5**).

Due to the popularity of the last presented method, we decided to check the protocol of synthesis of (1*E*,3*E*)-1,4-dinitro-1,3-butadiene (**1**) based on Scheme 6. In order to obtain 1,4-dinitrobutane-2,3-diol (**3**), a condensation reaction between nitromethane (**4**) and glyoxal (**5**) (Scheme 7) was applied [35,39,40,41]. 

**Scheme 8:**
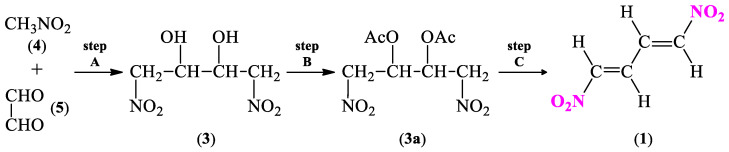
The sequence of transformations leading to the synthesis of (1*E*,3*E*)-1,4-dinitro-1,3-butadiene (**1**), carried out in this study.

The full path of obtaining (1*E*,3*E*)-1,4-dinitro-1,3-butadiene (**1**), including all synthesis steps which were carried out, is presented in Scheme 8.

In the first stage of the synthesis of nitrodiene **1** (Scheme 8, step A), a condensation reaction between nitromethane (**4**) and glyoxal (**5**) (Scheme 7) was carried out. In the literature the condensation has been described by four separate authors, with slight variations in preparatory procedures [35,39,40,41]. The differences were in the acidic and basic agents used (Table 1). Taking into account both acidic and basic factors as well as the yield, it was decided to synthesize nitrodiol **3** using the modified method presented by Novikov et al. [39]. The only change that was made was to use glacial acetic acid instead of SO_2_ as the acidic agent. Invariably, NaOH was used as the basic agent. It was this stage of synthesis of (1*E*,3*E*)-1,4-dinitro-1,3-butadiene (**1**) which proved to be the most problematic. 

Aqueous solutions of 35% NaOH and 40% glyoxal (**5**) were simultaneously added dropwise to an equivolume mixture of nitromethane (**4**) and methanol; the temperature was maintained at 0–2 °C. The mixture was maintained at 0 °C for one more hour, acidified with 50.0% solution of acetic acid in water, until a pH of 5 was reached. The organic phase was separated and the inorganic one was extracted four times with nitromethane as described by Novikov et al. [39]. 

The solvent from extracts was evaporated, the crystals washed with a minimal amount of water, yielding 15.5%. Novikov et al. [39] claim that the process yields 80.5% of nitrodiol’s **3** stereoisomers combined. The yield obtained by modification of the procedure was improbable and unsatisfying. Another series of four extractions with nitromethane followed, the evaporation of the extracts from the second round yielded further 16.8% of compound **3**. The total yield after primal and additional extraction equated to 32.3% which was still lower than 80.5% claimed by Novikov et al. [39].

Therefore, an attempt to obtain diol **3** by the method described by Plaut [41] was undertaken. During the synthesis it was found that leaving the reaction mixture unacidified overnight (as proposed by Plaut [41] in the example I) and acidifying it with acetic acid the next day led to unexpected self-heating of the mixture an hour after the addition of the acidic agent. The mixture heated itself to about 40 °C which lead to the complete disappearance of diol **3** (as controlled by TLC eluent cyclohexane–ethyl acetate CyH:EtOAc 80:20 *v*/*v*, developed with iodine).

The condensation of nitromethane (**4**) and glyoxal (**5**) was once again conducted similarly as described by Novikov et al. [39] with the substitution of SO_2_ to acetic acid until the pH reached 5. KOH was also used instead of NaOH, as proposed by Carroll and collaborators [40,42], in an effort to increase the reaction yield, as the authors used KOH and claimed good and probable results. The time of the reaction after the addition of all substrates was also greatly reduced, and the mixture was diluted and acidified right after the substrates were added. This treatment prevented the darkening of the reaction mixture. The acidified mixture was divided, and a few approaches for the extraction of 1,4-dinitrobutane-2,3-diol (**3**) were tested.

Next, an effort to find an alternative method of extraction was undertaken. After the acidification of acetic acid until pH 5, the mixture split into two phases, and they were separated. The aqueous phase was extracted once with either nitromethane, toluene, or diethyl ether separately. The process was monitored by the TLC technique (eluent cyclohexane–ethyl acetate CyH:EtOAc 80:20 *v*/*v*, developed with iodine). During the research it was found that neither of the tested solvents were appropriate; diol 3 was not present in the organic extracts. It was rationalized that due to the glycolic nature of the diol **3**, extraction from the aqueous phase might be a problematic stage. Therefore, the aqueous phase was evaporated under a vacuum at 40 °C. The evaporation yielded a viscous, amber-colored liquid that formed crystals when cooled to −18 °C overnight. A priori, the evaporated mixture could contain diol **3**, potassium acetate, and colored substances. Samples of the dried mixture were washed with various solvents and the solubility of mixture’s ingredients was assessed (Table 2). Based on information in Table 2, it can be concluded that among all tested solvents, the diethyl ether is most suitable because it dissolved the diol, while the colored fraction as well as potassium acetate were practically insoluble. 

Based on the conducted research, a protocol for extracting diol **3** was proposed and tested. For this purpose, the viscous amber concentrate was mixed with anhydrous sodium sulphate, so that the mixture had a consistency of wet sand. The mixture was covered and set aside for 30 min. After that time, the solid was transferred into a Soxhlet extractor and extracted with diethyl ether under an inert gas. After the extraction, the extract was cooled, solid that had fallen out was separated and washed sparingly with diethyl ether. The filtrate was evaporated dry on a rotatory evaporator in a bath of 40 °C. The dry crystals were washed with diethyl ether. In total, the isomer mixture of the 1,4-dinitro-2,3-butanediol (**3**) (44.5%) was obtained in the form of white lumpy and needle-like crystals. 

Definitely, the hardest challenge for the presented experimental part of study was the step A (Scheme 8) of obtaining 1,4-dinitro-2,3-butanediol (**3**). Both of the 2,3-diacetoxy-1,4-dinitrobutane (**3a**) as well as (1*E*,3*E*)-1,4-dinitro-1,3-butadiene (**1**) synthesis were carried out based on the procedures described by Novikov et al. [39]. The synthesis protocols both of obtaining ester **3a** and nitrodiene **1** (step B and C, Scheme 8) are not modified. All procedures are collected and presented in Section 3.1 “Materials and Methods”.

In order to obtain 2,3-diacetoxy-1,4-dinitrobutane (**3a**) (step B, Scheme 8), the acetyl chloride was applied in glacial acetic. The mixture was heated under reflux until the end of the release of hydrogen chloride gas. This moment was monitored through clouding of a drop of 3.0% solution of AgNO_3_ under the influence of HCl fumes. Before proceeding to the last stage, the obtained ester **3a** was thoroughly dried in a desiccator over phosphorus pentoxide under vacuum until the powder. The (1*E*,3*E*)-1,40-dinitro-1,3-butadiene (**1**) (step C, Scheme 8) was synthesized in thermal dehydro-acetylation using potassium bicarbonate in chloroform as the reaction solution.

#### 2.1.2. Spectral Characteristics

In order to confirm the structure of the obtained compound as well as to supplement the information missing in the literature about (1*E*,3*E*)-1,4-dinitro-1,3-butadiene (**1**), the spectral analyses such as UV-Vis, IR, ^1^H NMR, ^13^C NMR and 2D ^1^H-^13^C HMQC NMR were performed.

In particular, the UV-Vis, showed in Figure S1, confirmed [33,40] the presence of only one maximum absorption in a range of 500–190 nm, which is located at λ = 281 nm (CH_3_OH). In turn, IR spectrum, presented in Figure S2, allowed for finding the signals coming from ~C–H fragments (3106 and 3059 cm^−1^), nitro groups (1502 and 1342 cm^−1^) as well as fragments characteristic for conjugated alkene (1606 cm^−1^) connections. Signals coming from the *trans* alkene (1749 and 985 cm^−1^) fragments also turned out to be significant for analysis. 

The analysis of the ^13^C NMR spectrum, shown in Figure S4, indicates that the carbon C1 and C4 atoms, directly connected with nitro groups, are strongly shifted towards the weaker area to δ = 146.60 ppm. In turn, the signal δ = 129.50 ppm can be assigned to the central C2 and C3 carbon atoms. On the other hand, thanks to the analysis of ^1^H NMR (Figure S3) together with the analysis of 2D ^1^H-^13^C HMQC NMR (Figure S5), it was possible to assign signals to all hydrogen atoms in (1*E*,3*E*)-1,4-dinitro-1,3-butadiene (**1**).

#### 2.1.3. Understanding the Geometric Isomerism Based on DFT Calculations

**Scheme 9:**
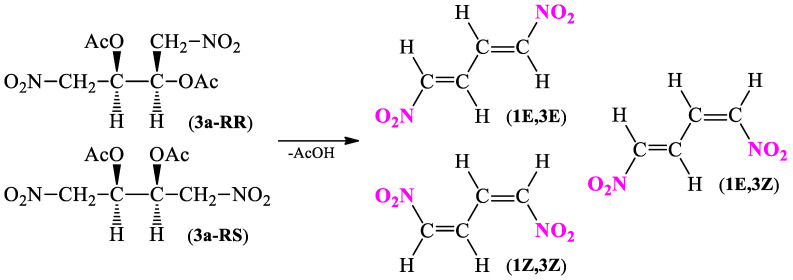
Possible substrates and products of the dehydro-acetylation reaction of 2,3-diacetoxy-1,4-dinitrobutane (**3a**) to 1,4-dinitro-1,3-butadiene (**1**).

In 1970, Durden et al. [35] reported that dinitrodiene **1** occurs in the conformation of (1*E*,3*E*). In order to confirm the presented hypothesis, a comprehensive analysis of the dehydro-acetylation reaction of 2,3-diacetoxy-1,4-dinitrobutane (**3a**) to 1,4-dinitro-1,3-butadiene (**1**) (Scheme 9) using quantum chemical tools was performed.

For this purpose, the computational calculation using B3LYP/6-31G(d) model in a gas phase was used. Firstly, the optimization process of two forms (*RR*) and (*RS*) of 2,3-diacetoxy-1,4-dinitrobutane (**3a**) as well as three forms (1*E*,3*E*), (1*E*,3*Z* = 1*Z*,3*E*), (1*Z*,3*Z*) of 1,4-dinitro-1,3-butadiene (**1**), and acetic acid molecules was conducted. Then, the thermodynamic properties of all possible combinations of decomposition reactions (Scheme 9) were calculated (Table 3).

According to the results presented in Table 3 it can be observed that regardless of the isomer of 2,3-diacetoxy-1,4-dinitrobutane (**3a**), the more favored product for dehydro-acetylation reaction of ester **3a** from the thermodynamic point of view is (1*E*,3*E*)-1,4-dinitro-1,3-butadiene (**1**). The conclusion stands in agreement with observations made by Durden et al. [35] and with the results of the IR analysis.

### 2.2. Study of Electron Density Distribution, Bioactivity and Pharmacokinetics Indices Based on MEDT, ADME and PASS

The computational study has been conducted for (1*E*,3*E*)-1,4-dinitro-1,3-butadiene (**1**) and its simpler analogue *S*-trans-1,3-butadiene (**1′**) to show changes in the structure of the molecule introduced by the presence of the nitro group. The obtained results have been divided into four parts. Firstly, a topological analysis of Electron Localization Function (ELF) together with Natural Population Analysis (NPA), and Molecular Electrostatic Potential (MEP) for molecules **1** and **1′** at the ground states (GS) has been performed in order to characterize their electronic structures. Secondly, an analysis of reactivity indices based on Conceptual Density Functional Theory (CDFT) for compounds **1** and **1′** at the ground state has been carried out. In the next part, the Non-Covalent Interactions (NCI) analysis is performed in order to obtain a deeper insight of the structure of molecules **1** and **1′**. Finally, a comprehensive evaluation of the chemical structure of (1*E*,3*E*)-1,4-dinitro-1,3-butadiene (**1**) has been conducted to determine the likeness of nitrodiene **1** activity as a pharmaceutic. To assess the potential biological activity, an in silico protocol of Absorption, Distribution, Metabolism, Excretion (ADME) has been used. In order to better research the biological significance of nitrodiene **1,** a simple protocol of Prediction of Activity Spectra for Substances (PASS) was also carried out.

#### 2.2.1. Analysis of the Electronic Structure of the Molecules **1** and **1′** Based on ELF, NPA and MEP

**Figure 2:**
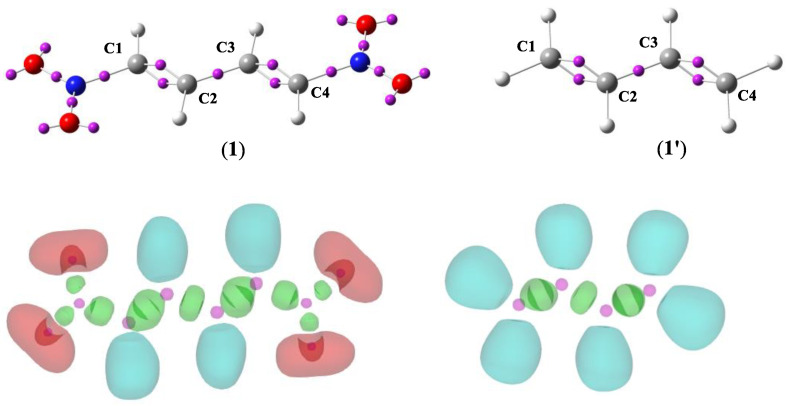
B3LYP/6-31G(d) ELF attractor positions of the core and valence basins for (1*E*,3*E*)-1,4-dinitro-1,3-butadiene (**1**) and *S-trans*-1,3-butadiene (**1′**) together with ELF localization domains of the compounds represented at an isosurface value of ELF = 0.75. For ELF localization domains, protonated basins are shown in blue, monosynaptic basins in red, disynaptic basins in green, and core basins in magenta. In the first row, the ELF attractors are shown as pink spheres.

The ELF is a technique which allows for the prediction of the likelihood of finding an electron in the neighboring space of a reference electron, at a given point and with the same spin. The technique is based on electron density distribution in molecules and allows both to characterize the electronic structures of compounds, and to predict their reactivity in chemical processes [43]. The ELF attractor positions of the core and valence basins together with ELF localization domains for (1E,3E)-1,4-dinitro-1,3-butadiene (**1**) and S-trans-1,3-butadiene (**1′**) are shown in Figure 2, while the most relevant valence basin populations are given in Table 4.

The ELF topology of *S-trans*-1,3-butadiene (**1′**) presents two pairs of disynaptic basins V(C1,C2) and V’(C1,C2) (Figure 2), integrating a total electron population of 3.44 e (Table 4) as well as V(C3,C4) and V’(C3,C4) (Figure 2), integrating the same value of total electron population of 3.44 e (Table 4). The presence of these pairs is associated with somewhat depopulated C1-C2 and C3-C4 double bonds. In turn, the presence of disynaptic basin V(C2,C3) (Figure 2), integrating a population of 2.30 e (Table 4), is associated with a C2-C3 single bond. 

**Figure 3:**
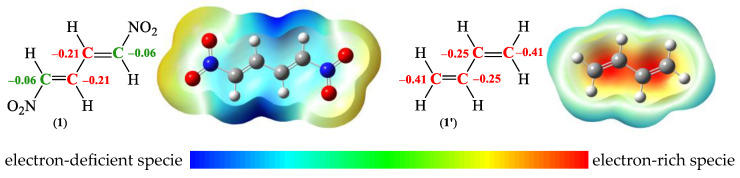
B3LYP/6-31G(d) proposed ELF-based Lewis-like structures with the natural atomic charges for (1*E*,3*E*)-1,4-dinitro-1,3-butadiene (**1**) and *S-trans*-1,3-butadiene (**1′**) together with the molecular electrostatic potential maps. Negative charges are colored in red, while negligible charges are colored in green. Natural atomic charges are given an average number of electrons [e].

In turn, the ELF topology for (1*E*,3*E*)-1,4-dinitro-1,3-butadiene (**1**) is practically identical. When compared with the *S-trans*-1,3-butadiene (**1′**), the total electron population associated with the presence of two double bonds C1-C2 as well as C3-C4 is slightly higher and the total electron population associated with the presence of a C2-C3 single bond is slightly smaller (Table 4). Therefore, the introduction of two symmetrically located nitro groups to the structure does not affect the electron localization. Based on ELF analysis, the proposed Lewis-like structures together with the natural atomic charges for compounds **1** and **1′** are given in Figure 3.

The charge distribution for molecules **1** and **1′** was examined through NPA [44,45]. The analysis indicates a negative distribution located at the central carbon atoms C2 and C3 which form a single bond. For *S-trans*-1,3-butadiene (**1′**), both natural atomic charges are -0.25 e (Figure 3). In the case of (1*E*,3*E*)-1,4-dinitro-1,3-butadiene (**1**) for analogous carbon atoms C2 and C3, a slight decrease in electron-rich species to -0.21 e is noticeable (Figure 3). A different situation is observed in the case of terminal carbon atoms. Both C1 and C4 carbon atoms in diene **1′** are represented by highly electronegative region, −0.41 e (Figure 3). On the other hand, the nitro groups introduced to nitrodiene **1** strongly interact with C1 and C4 carbon atoms. As a result, these centers are depleted of electrons to a value typical of neutral regions, 0.06 e (Figure 3). The presented results correlate well with MEP [46]. The analysis of a surface shows a highly negative electrostatic potential around all carbon atoms in diene **1’** (in red) and poorly electropositive region within the vicinity of the hydrogen atoms (in green-blue). In turn, in nitrodiene **1,** a positive electrostatic potential is found both around the carbon atoms (in light-blue) as well as a stronger electropositive region within the vicinity of the hydrogen atoms (Figure 3).

#### 2.2.2. Analysis of the CDFT Reactivity Indices for the Compounds **1** and **1′**

**Figure 4:**
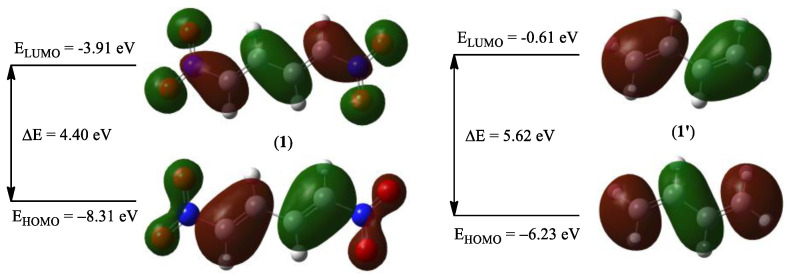
B3LYP/6-31G(d) HOMO-LUMO energy gap diagram for (1*E*,3*E*)-1,4-dinitro-1,3-butadiene (**1**) and *S-trans*-1,3-butadiene (**1′**).

CDFT is a very important tool in understanding the reactivity of molecules in polar processes. The theory is to connect well-established chemical concepts—like ionization potential l, electron affinity A, electronic chemical potential μ, Mulliken electronegativity X, chemical hardness η, and chemical softness S—with the electronic structure of a molecule. Based on those, the indication of global electronic properties of substrates, such as global electrophilicity ω, and global nucleophilicity N for molecules, can be established. As an effect, it is possible to assign addends a role of either an electrophile or a nucleophile in the studied reactions [47,48,49]. Furthermore, with the application of Parr functions, not only global, but also local electronic properties of a molecule can be estimated, thus allowing for the prediction of reactivity of molecules in the studied reactions, based only on substrates structures [50,51]. The global reactivity indices for (1*E*,3*E*)-1,4-dinitro-1,3-butadiene (**1**) and *S-trans*-1,3-butadiene (**1′**) are given in Table 5, while the local electronic properties are shown in Figure 4.

The computed HOMO energy [47] of *S-trans*-1,3-butadiene (**1′**) is −6.23 eV and the LUMO energy [47] for diene **1′** is 0.61 eV (Table 5). In turn, the presence in structure two shows strongly electron-withdrawing nitro groups in (1*E*,3*E*)-1,4-dinitro-1,3-butadiene (**1**) which causes a reduction of both HOMO energy, to -8.31 eV, and LUMO energy, to 3.91 eV (Table 5). Consequently, the HOMO-LUMO energy gap shrinks. For diene **1’** it equates to 5.62 eV and for nitrodiene **1** it is 4.40 eV (Figure 4). The HOMO-LUMO energy gap is an important stability index as it explains the charge transfer interactions within the molecule and is also useful in determining molecular electronic transport properties. A molecule with a high frontier orbital HOMO-LUMO energy gap has a low chemical reactivity and simultaneously high kinetic stability. The phenomena is related with high excitation energy between the high-lying LUMO and the low-lying HOMO energy levels [52,53,54]. The estimated energy gaps mean that the (1*E*,3*E*)-1,4-dinitro-1,3-butadiene (**1**) is less stable compared to S-trans-1,3-butadiene (**1′**) and that the nitrodiene **1** is characterized with higher reactivity in chemical reactions compared to diene **1′**.

**Figure 5:**
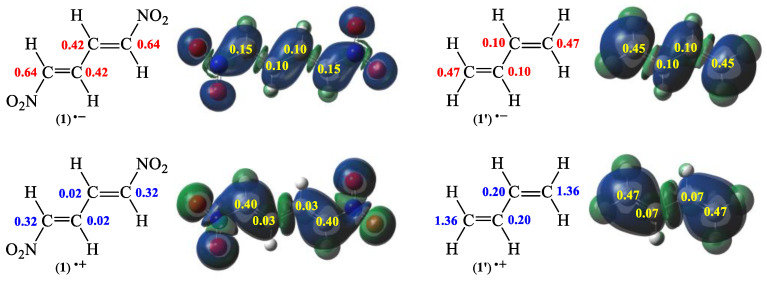
B3LYP/6-31G(d) local electronic properties of diene **1′** and nitrodiene **1** presented as three-dimensional representations (3D) of Mulliken atomic spin densities for radical anions **1˙^−^** and **1′˙^−^** and radical cations **1˙^+^** and **1′˙^+^** together with the electrophilic P_k_^+^ and the nucleophilic P_k_**^−^** Parr functions values (given in yellow) as well as the indices of the local electrophilicity ω_k_ of **1˙^−^** and **1′˙^−^** (given in red, in eV) and the local nucleophilicity N_k_ of **1˙^+^** and **1′˙^+^** (given in blue, in eV).

The calculated global electrophilicity [55] ω index of *S-trans*-1,3-butadiene (**1′**) is 1.04 eV and the calculated global nucleophilicity [56] N index for this diene (**1′)** is 2.89 eV (Table 5). These values give the conclusion that diene **1′** can be classified as moderate electrophile as well as moderate nucleophile in polar reactions, within the electrophilicity and nucleophilicity scale [47]. In turn, the introduction of two strongly electron-withdrawing nitro groups to the molecule, in (1*E*,3*E*)-1,4-dinitro-1,3-butadiene (**1**), completely changes the preference of reactivity indices. In particular, the calculated global electrophilicity [55] ω index is significantly increased to 4.24 eV and the calculated global nucleophilicity [56] N index is significantly reduced to 0.81 eV (Table 5). These values give the conclusion that nitrodiene **1** can be classified as an extremely strong electrophile and marginal nucleophile in a polar reaction, within the electrophilicity and nucleophilicity scale [47]. The presented information is an important conclusion, as in the reactions, including the participation of non-symmetric reagents; the regioselectivity can be defined through interaction between the most electrophilic center of the electrophile and the most nucleophilic center of the nucleophile [50]. To characterize the most nucleophilic and the most electrophilic centers of (1*E*,3*E*)-1,4-dinitro-1,3-butadiene (**1**) and *S-trans*-1,3-butadiene (**1′**), the electrophilic P_k_^+^ and nucleophilic P_k_^−^ Parr functions together with local electrophilicity ω_k_ and local nucleophilicities N_k_ of diene **1′** and nitrodiene **1** were analyzed (Figure 5) [51].

The analysis of the electrophilic P_k_^+^ Parr function [51] of *S-trans*-1,3-butadiene (**1′**) indicates that the terminal carbon atoms are the most electrophilic centers of this species, both presenting the value P_C_^+^ = 0.45. At these atoms, the value of the local electrophilicity ω_k_ index is ω_C_ = 0.47 eV (Figure 5). In turn, electrophilic P_k_^+^ Parr functions for the centric atoms of diene **1′** are visibly reduced to P_C_^+^ = 0.10 (ω_C_ = 0.10 eV) (Figure 5). Analysis of the electrophilic P_k_^+^ Parr function of (1*E*,3*E*)-1,4-dinitro-1,3-butadiene (**1**) show a similar tendency. In particular, the most electrophilic centers in this molecule **1** are situated on the terminal carbon atoms, presenting the value P_C_^+^ = 0.15. At these atoms, the value of the local electrophilicity ω_k_ index is ω_C_ = 0.64 eV (Figure 5). In turn, the values of electrophilic P_k_^+^ Parr function for the centric atoms of nitrodiene **1** are slightly reduced to P_C_^−^ = 0.10 (ω_C_ = 0.42 eV) (Figure 5). Based on the presented analysis, it can be concluded that the direct neighborhood of strongly electron-withdrawing nitro groups causes a significant reduction of local electrophilic properties in dinitrodiene **1** when compared to diene **1′**. 

On the other hand, analysis of the nucleophilic P_k_^−^ Parr functions [51] of *S-trans*-1,3-butadiene (**1′**) indicates that the terminal carbon atoms are also the most nucleophilic centers of this species, presenting the values P_C_^−^ = 0.47. At these atoms, the values of the local nucleophilicity N_k_ index is N_C_ = 1.36 eV (Figure 5). In turn, values of nucleophilic P_k_^−^ Parr functions for the centric atoms of diene **1′** are extremely reduced to P_C_^−^ = 0.07 (N_C_ = 0.20 eV) (Figure 5). The differences between nucleophilic properties for nonsymmetric atoms in molecule **1′** are slightly greater than for electrophilic properties. Another situation is observed in the case of (1*E*,3*E*)-1,4-dinitro-1,3-butadiene (**1**). Analysis of the nucleophilic P_k_^−^ Parr function of nitrodiene **1** shows that the most nucleophilic centers in molecule **1** are situated on the terminal carbon atoms, presenting the value P_C_^−^ = 0.40. At these atoms, the values of the local nucleophilicity N_k_ index is N_C_ = 0.32 eV (Figure 5). In turn, values of nucleophilic P_k_^−^ Parr function for the centric atoms of nitrodiene **1** are reduced practically to zero, P_C_^−^ = 0.03 (N_C_ = 0.02 eV) (Figure 5). Based on the presented analysis, it can be concluded that the direct neighborhood of a strongly electron-withdrawing nitro group does not cause a significant reduction in local nucleophilic properties of nitrodiene **1** in comparison to diene **1′**.

#### 2.2.3. Analysis of the Non-Covalent Interactions in the Molecules **1** and **1′** Based on NCI

**Figure 6:**
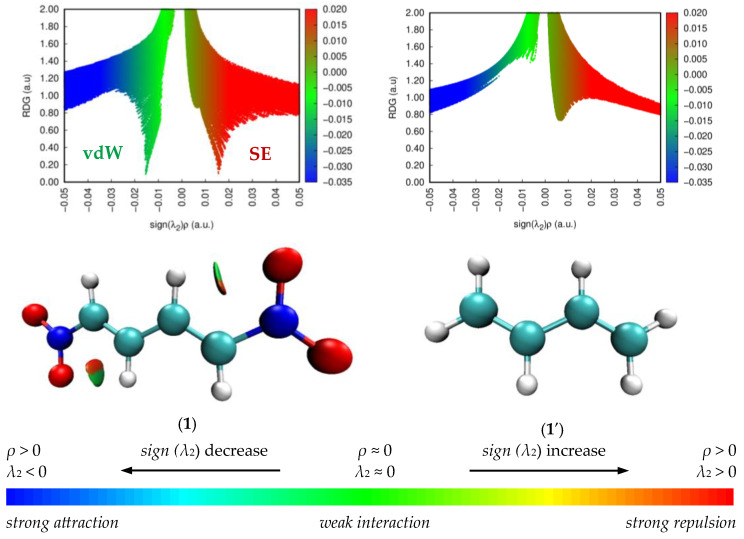
B3LYP/6-31G(d) non-covalent interaction scatter diagram together with the reduced density gradient (RDG) analysis for diene **1′** and nitrodiene **1**. The strong attractive interactions like H-bond or halogen-bond are colored in blue, the van der Waals interactions are colored in green while the repulsive or steric interactions are colored in red.

The NCI is a very useful and popular method for revealing the location and types of weak interactions in chemical systems. The analysis is related to the electron density (ρ), and the reduced density gradient (s). Due to the size of the molecules, the most common approach is an assignment of van der Waals interactions (vdW), steric effects (SEs), and hydrogen bonds (HBs) based on pairwise distances between atoms according to their vdW radii. In an effect, a direct representation and characterization of non-covalent interactions in three-dimensional space is possible. Therefore, NCI is a useful tool in understanding many chemical, biological, and technological problems [57,58]. A non-covalent interaction scatter diagram together with a reduced density gradient (RDG) analysis for diene **1′** and nitrodiene **1** is presented in Figure 6. A negative sign indicates attractive forces connected with hydrogen and halogen bonding and is marked in blue color; van der Waals forces are colored green, whereas red fragments, to the right from zero, express the repulsive forces, which mostly occur in steric interactions as well as in the presence of rings.

The NCI analysis of *S-trans*-1,3-butadiene (**1′**) indicates that molecule **1′** does not have any characteristic non-covalent interactions (Figure 6). A completely different situation occurs in the case of (1*E*,3*E*)-1,4-dinitro-1,3-butadiene (**1**). The NCI analysis shows that the introduction of two strongly electron-withdrawing nitro groups significantly affect the intramolecular interactions in molecule **1**. Particularly in the area between the nitrogen atom, the terminal carbon atom and the centric carbon atom (O_2_N−CH=CH− fragment) an intramolecular interaction, located symmetrically on both sides of the molecule, is observed. The occurrence of these interactions is related to two types of non-covalent effects. The first of them is a steric interaction (part of the red-colored area, sign (λ_2_)*ρ* between 0.02 and 0.01 a.u.) (Figure 6). The second component relates to a van der Waals interaction (part of the green-colored area, sign (λ_2_)*ρ* between −0.02 and −0.01 a.u.). It can be identified with an interaction of the oxygen atom of the nitro group and the hydrogen atom in the diene part (Figure 6).

#### 2.2.4. Analysis of Drug-Likeness and ADME Studies of the Molecule **1**

ADME studies are critical in modern drug discovery [59]. The process of predicting a good drug by analyzing its pharmacokinetic properties in the laboratory is expensive, and labor-intensive. So, to accelerate the research and more rationally dispense financing, ADME parameters can be inspected using in silico tools. The undoubted advantage of in silico ADME studies is the fact that the analysis is based on the structure of the compound, including the atoms’ presence in the molecule and their connections. Therefore, ADME is one of the simplest and most useful methodologies for the initial examination of drug-likeness [60,61]. Within the in silico ADME properties study for (1*E*,3*E*)-1,4-dinitro-1,3-butadiene (**1**), some physicochemical properties like lipophilicity, water solubility, pharmacokinetics and also medicinal chemistry assessments were performed by an online server (SwissADME [59]) and are summarized in Table 6. 

According to the obtained parameters, the assessment using models developed by Lipinski et al. [62], Ghose et al. [63], Veber et al. [64], Egan et al. [65] and Muegge et al. [66] was performed to evaluate the drug-likeness. The main features and conditions of these rules and filters are collected in Table 7.

Based on physicochemical descriptors, the predicted ADME parameters shown in Table 6, and the main features of the five drug-likeness rules shown in Table 7, it can be concluded that (1*E*,3*E*)-1,4-dinitro-1,3-butadiene (**1**) is a good drug candidate according to the filters by Lipinski et al. [62], Veber et al. [64], and Egan et al. [65]. The compound **1** has an appropriate molecular weight, number of rotatable bonds, H-bond donor–acceptor ratio, and satisfactory value of topological polar surface area (TPSA), which is a commonly used parameter for the optimization of a drug’s ability to permeate cells [67]. What is more, dinitrodiene **1** has excellent lipophilicity properties.

However, according to more demanding filters such as those by Ghose et al. [63] and Muegge et al. [66], (1*E*,3*E*)-1,4-dinitro-1,3-butadiene (**1**) has low potential as a drug candidate. Both of the filters define a minimum molecular weight. Nitrodiene **1** does not meet their criteria due to its low weight and molar refractivity [68]. Nitrodiene **1** has only 14 atoms in total, only four of which are carbon atoms.

The analysis of other drug-likeness parameters (Table 6), not included in the tested filters, shows that (1*E*,3*E*)-1,4-dinitro-1,3-butadiene (**1**) not only has good lipophilic properties but is also water-soluble. These physicochemical properties are crucial to drugs’ uptake and their further metabolism [69,70]. What is more, nitrodiene **1** is not one of the Pan-Assay Interference Compounds (PAINS) but due to its structure it causes two alerts, due to the presence of a nitro group and the absence of an organic ring. Additionally, based on the data shown in Table 6 it should be concluded that the (1*E*,3*E*)-1,4-dinitro-1,3-butadiene (**1**) can have good gastrointestinal (GI) absorption and does not inhibit any of the presented cytochrome P450 isoforms, which are significant in drug elimination through the process of metabolic biotransformation. On the other hand, nitrodiene **1** does not easily permeate the blood–brain barrier. The logarithm of skin permeation coefficient for nitrodiene **1** is −6.60 cm/s (Table 6).

**Figure 7:**
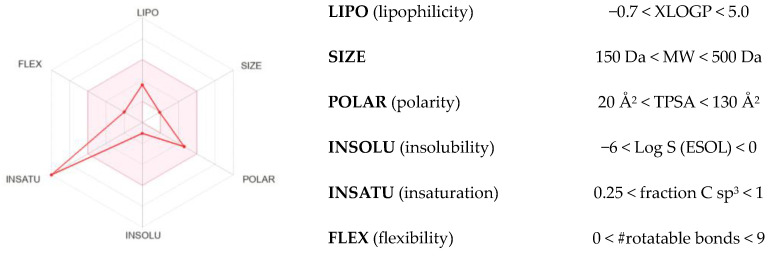
The bioavailability radar of the drug-likeness for (1E,3E)-1,4-dinitro-1,3-butadiene (**1**). The pink area represents the optimal range for each property like lipophilicity, size, polarity, insolubility, insaturation and flexibility.

Finally, the bioavailability radar which allows us to visually assess the drug-likeness of the nitrodiene **1** is presented in Figure 7. Based on the performed analysis, it can be concluded that the main problem presented in the bioavailability radar is total instauration in the molecule of nitrodiene **1**. It is caused by the sp^2^ hybridization of all the carbon atoms. It should be underlined that the optimal ratio of carbon atoms in the sp^3^ hybridization to total carbon atoms ranges between 0.25 and 1 [60,61].

#### 2.2.5. Assessment of Antimicrobial Activities Based on PASS for the Molecule **1**

At the end, the antimicrobial activity of (1*E*,3*E*)-1,4-dinitro-1,3-butadiene (**1**) was predicted by applying PASS which is a component of the Way2Drug portal [71]. The PASS is a simple and very useful tool that calculates the potential biological activity of a molecule and allows for the preliminarily assessment of a molecule’s drug-likeness, possible mechanisms of its action, pharmacological and adverse effects, toxicity, and other parameters. The assessment is made based on the molecule’s structural formula. The results of the PASS analysis are expressed as a molecule’s probabilities to be active (Pa) or inactive (Pi). The values of Pa and Pi range from 0.000 to 1.000, where the value of 0.000 indicates a complete lack of probability, while a value of 1.000 means certainty. The compound can be assigned as probably active when Pa > Pi. The obtained data is useful to decide (I) if the synthesis and characterization of new compounds is justified prior to preparing them; and (II) if the screened compounds are good candidates for further biological research [72]. In order to evaluate primary biological properties of nitrodiene **1,** an assessment of antimicrobial activities was performed by the online server PASS [71] and summarized in Table 8.

According to the PASS analysis presented in Table 8, (1*E*,3*E*)-1,4-dinitro-1,3-butadiene (**1**) has a very low antimicrobial activity. All Pa parameters against microorganisms, namely antiviral, antifungal, antibacterial as well as antiparasitic are lower than 0.6. To deem a compound potentially active, the Pa parameter should be higher than 0.7 [73]. However, nitrodiene **1** shows partial biological activity. For example, compound **1** can be tested in a role of an inhibitor against enzymes of *Saccharopepsin* (Pa = 0.912), *Acrocylindropepsin* (Pa = 0.912), *Polyporopepsin* (Pa = 0.864) or applied in the treatment of pancreatic disorders (Pa = 0.826) as well as many others. The full information about the biological activity and potential application directions of nitrodiene **1** (Pa > 0.7) is collected in Table S3 in Supplementary Materials. It should be underlined that in all cases, Pa > Pi. Therefore, the mentioned activities are possible.

## 3. Materials and Methods

### 3.1. Materials

Commercially available (Sigma–Aldrich, Szelągowska 30, 61-626 Poznań, Poland) reagents and solvents were used. All solvents were tested with high-pressure liquid chromatography before use.

### 3.2. General Procedure for 1,4-Dinitro-2,3-butanediol *(**3**)* Isomers Synthesis

First, 35 cm^3^ of nitromethane (**4**) and 35 cm^3^ of methanol were added to a flask and stirred. Then, everything was cooled to a temperature of 0 °C. Next, 5.8 cm^3^ of 40% glyoxal aqueous solution (**5**) as well as a solution of 6.4 g KOH in 6.0 cm^3^ distilled water was added drop by drop to the cold mixture in an alternating way, maintaining the temperature below 5 °C all the time. Immediately after the glyoxal and KOH solution, 25 cm^3^ of distilled water was added to the flask, the mixture was neutralized by 12.0 cm^3^ of glacial acetic acid, to pH = 5. At the end, the mixture was delivered to an ambient temperature.

After the reaction, the post-reaction mixture was concentrated by vacuum evaporator. The obtained concentrate has a form of thick syrup, slowly darkening at room temperature. When the concentrate must be stored it should be stored frozen at around −18 °C. Anhydrous Na_2_SO_4_ (5.0 g of Na_2_SO_4_ for 1.0 g of concentrate) was mixed with the concentrate and left covered for 30 min so that the inorganic salt could absorb the residual water. The final mixture should have the consistency of slightly damp sand. 

The solid was extracted with diethyl ether in a Soxhlet extractor, under an inert gas, for around 90 min. After extraction, the solvent was cooled. If the solid has precipitated from the diethyl ether, it should be separated by filtering and washing with a small amount of diethyl ether, the filtrate should be evaporated dry in a rotatory evaporator, and the obtained solids washed sparingly with diethyl ether. If not, the extract should be evaporated dry in a vacuum evaporator, and the solids washed sparingly with diethyl ether. In total, 4.0 g (44.5%) of the mixed isomers of the 1,4-dinitro-2,3-butanediol (**3**) was obtained in the form of white lumpy and needle-like crystals. The melting points of the products are 87.5–88.0 °C and 92.5–93.0 °C, respectively.

### 3.3. General Procedure for 2,3-Diacetoxy-1,4-dinitrobutane *(**3a**)* Isomers Synthesis

First, 7.7 g of the isomer mixture of 1,4-dinitro-2,3-butanediol (**3**), 19.3 cm^3^ of acetyl chloride and 95 cm^3^ of glacial acetic acid were to a flask added and stirred. Then, all was heated under reflux until the end of the release of hydrogen chloride gas. This moment was monitored through clouding of a drop of 3.0% solution of AgNO_3_ under the influence of HCl fumes escaping the condenser. Next, the heating was stopped, and the mixture was delivered to an ambient temperature. After cooling, the mixture was added to 400 g of finely crushed ice. When the ice completely melted, the obtained solid was ground under the liquid, then the mixture was filtered and the solid washed sparingly with water. The wet ester was dried in a desiccator over phosphorus pentoxide under vacuum until the powder was completely dry (when tested with a cobalt chloride test strip). In total, 9.2 g (81.0%) of the isomers mixture of the 2,3-diacetoxy-1,4-dinitrobutane (**3a**) was obtained in the form of a white dust. According to the measured melting point, the product was a mixture of two fractions. The melting points of those are 71.0–73.0 °C and 75.0–77.0 °C, respectively (after recrystallization from ethanol).

### 3.4. General Procedure for (1E,3E)-1,4-Dinitro-1,3-butadiene *(**1**)* Synthesis

First, 8.19 g of a mixture of 2,3-diacetoxy-1,4-dinitrobutane (**3a**) isomers, 0.3 g of KHCO_3_ and 230 cm^3^ of chloroform were added to a 500 cm^3^ flask equipped with a magnetic stirrer, a reflux condenser, and a heating mantle. The reagents were heated together under reflux for 4 h. After that, the post-reaction mixture was cooled and filtered under vacuum. The obtained sediment was washed with chloroform. Then, the filtrate was evaporated on a vacuum evaporator to obtain about 4.0 g of slightly moist, yellow crystals, smelling strongly of vinegar. The crystals should be washed with a small amount of distilled water to remove the rest of acetic acid. Immediately after rinsing, the crystals must be dried in a desiccator over phosphorus oxide, under vacuum and without access of light. In total, 3.0 g (65.0%) of (1*E*,3*E*)-1,4-dinitro-1,3-butadiene (**1**) was obtained in the form of yellow crystals smelling of freshly cut cedar wood.

(1*E*,3*E*)-1,4-dinitro-1,3-butadiene (**1**): m.p. 146.5 °C; UV-Vis (CH_3_OH): λ_max_ [nm] 281; FT-IR (ATR): υ [cm^−1^] 3106 and 3059 (~C–H stretch, alkene, medium), 1749 (stretch, *trans* alkene, weak); 1606 (>C=C< stretch, conjugated alkene, medium), 1502 (~N–O stretch, asymmetrical, nitro group, strong), 1342 (~N–O stretch, symmetrical, nitro group, strong), 985 (>C=C< bend, *trans* alkene, strong); ^1^H NMR (400 MHz, CDCl_3_): δ [ppm] 7.63 (d, 1H, CH–NO_2_, *J* = 9.6 Hz), 7.63 (dd, 1H, =CH–, *J*_1_ = 3.2 Hz, *J*_1_ = 9.6 Hz), 7.48 (dd, 1H, =CH–, *J*_1_ = 3.2 Hz, *J*_1_ = 9.7 Hz), 7.47 (d, 1H, =CH–NO_2_, *J* = 9.7 Hz); ^13^C NMR (100 MHz, CDCl_3_): δ [ppm] 146.60 (C1 and C4); 129.50 (C2 and C3).

### 3.5. Analytical Techniques

For reaction progress testing, thin layer chromatography (TLC) was performed using aluminum plates with silica (unmodified layers) as the standard procedure in the case of nitrogen-containing organic compounds [74,75,76]. As the role of an eluent, cyclohexane–ethyl acetate mixture CyH:EtOAc (80:20 *v*/*v*) was applied. The plates were developed by iodine treatment. The melting points were determined with the Boetius PHMK 05 apparatus and were not corrected. FT-IR spectra were derived from the FTS Nicolet IS 10 spectrophotometer with Attenuated Total Reflectance (ATR). The ^1^H NMR (400 MHz) and ^13^C NMR (100 MHz) spectra were recorded with a Bruker AVANCE NMR spectrometer. All spectra data were obtained in the deuterated chloroform CDCl_3_ (visible at 7.27 ppm for ^1^H NMR and at 77.00 ppm for ^13^C NMR) solutions and the chemical shifts (δ) are expressed in ppm, while the J-couplings (J) are given in Hz. TMS was used as an internal standard. UV-Vis spectra were determined in methanolic solutions for the 190–500 nm range with a spectrometer UV-5100 Biosens. The maximum of absorption was detected below 1 AU of absorbance and adheres to the Beer-Lambert law.

### 3.6. Computational Details

All computations were performed using the Gaussian 16 package [77] in the Ares computer cluster of the CYFRONET regional computer center in Cracow. DFT calculations were performed using the B3LYP functional [78] together with 6-31G(d) basis set [79]. This computational level is required by the MEDT approach [36,47] and has already been successfully used in optimization and evaluation of a various organic molecules [80], especially conjugated nitroorganic systems [81,82,83]. What is more, this computational level correlates well with experimental results [84,85,86]. Calculations of all critical structures were performed at temperature T = 298 K and pressure *p* = 1 atm in a gas phase. All localized stationary points were characterized using vibrational analysis. It was found that starting molecules as well as products had positive Hessian matrices. 

The electronic structures of (1*E*,3*E*)-1,4-dinitro-1,3-butadiene (**1**) and *S-trans*-1,3-butadiene (**1′**) were characterized by the Electron Localization Function (ELF) [43], the Natural Population Analysis (NPA) [44,45], the Molecular Electrostatic Potential (MEP) [46], and Non-Covalent Interactions (NCI) [57,58]. Analyses of global electronic properties of reactants were performed according to Domingo’s recommendations [47,48,49]. Electrophilic Parr functions P_k_^+^ and nucleophilic Parr functions P_k_^−^ were obtained from the Atomic Spin Density (ASD) of the reagents’ radical ions [50,51]. The physicochemical properties were evaluated by the SwissADME online server [59]. In order to assess drug-likeness, models based on rules of Lipinski et al. [62], Ghose et al. [63], Veber et al. [64], Egan et al. [65] and Muegge et al. [66] were applied. In turn, the analysis and prediction of activity spectra for the substances were prepared by the PASS online server [71].

The NPA, the ELF, as well as the NCI studies were performed with TopMod [87] and Multiwfn [88] software. For visualization of the molecular geometries of all structures and 3D representations of the radical anions and the radical cations, GaussView software [89] was used. In turn, the ELF localization domains were represented by using the Paraview software [90,91] at an isovalue of 0.75 a.u. The calculations of the NCI analysis were performed using the VMD [92] software and visualized at an isosurface value of 0.5.

## 4. Conclusions and Future Perspectives

In this research, a comprehensive study on the synthesis and spectral characteristics of (1*E*,3*E*)-1,4-dinitro-1,3-butadiene (**1**) was presented. Additionally, a computational analysis of electronic chemical parameters based on MEDT has been made. At the end, a forecasting study of the biological properties of nitrodiene **1** using ADME and PASS simulation was performed.

Among the available alternatives for the synthesis of (1*E*,3*E*)-1,4-dinitro-1,3-butadiene (**1**), a three-step method starting from nitromethane (**4**) and glyoxal (**5**) was selected in the presented study. Modifications of synthesis protocol were carried out in order to increase efficiency of obtaining the final nitrodiene **1,** and overall process safety. In particular, the modification of extraction method for the synthesis of 1,4-dinitrobutane-2,3-diol (**3**), led to greater yields of extracted product to ca. 40% as compared to 17% when the extraction protocol proposed by Novikov et al. [39] was applied. Furthermore, the new method uses less expensive diethyl ether instead of nitromethane. What is worth noting, the nitromethane is classified in category 2B as possibly carcinogenic to humans by the International Agency for Research on Cancer [93], whereas diethyl ether, among other volatile anaesthetics, is classified in category 3, which means it is not classifiable as to its carcinogenicity to humans [94]. According to NIOSH, the permissible exposure limits for diethyl ether and nitromethane are 400 ppm and 100 ppm, respectively [95]. Thus, the diethyl ether can be treated as a more favored solvent compared to nitromethane.

The structure of the obtained (1*E*,3*E*)-1,4-dinitro-1,3-butadiene (**1**) has been verified based on measured melting point and data available in the literature [35,38,39,42]. In order to further confirm the structure of the nitrodiene **1** as well as to supplement the information missing in the literature, spectral analyses such as UV-Vis, IR, ^1^H NMR, ^13^C NMR and 2D ^1^H-^13^C HMQC NMR were performed.

A comprehensive MEDT analysis of (1*E*,3*E*)-1,4-dinitro-1,3-butadiene (**1**) in comparison to *S-trans*-1,3-butadiene (**1′**) shows that the introduction of two nitro groups into the molecule causes changes in the electron density and its distribution parameters, but not in all studied aspects. The ELF analysis showed practically identical distribution of electron population for molecules **1** and **1’**, but the presence of nitro groups had a significant effect on charge distribution. In particular, the NPA and MEP analyses indicate drastic a increase in the natural atomic charges on terminal carbon atoms. Based on the analysis of CDFT, it can be concluded that nitrodiene **1** will play a role of a super strong electrophile and marginal nucleophile in polar reactions. The most electrophilic and nucleophilic center for nitrodiene **1** will be located on the terminal carbon atoms in direct neighborhood of the nitro groups. Finally, the NCI analysis presented that in the space between the nitrogen atom, the terminal carbon atom and the centric carbon atom (O_2_N−CH=CH− fragment) an intramolecular interaction, related to two types of non-covalent effects is observed. Its first component is a steric interaction while the second component relates to van der Waals interactions between the nitro group’s oxygen atom and the hydrogen atom in the diene part.

Based on physicochemical descriptors as well as predicted ADME and PASS parameters it can be concluded that (1*E*,3*E*)-1,4-dinitro-1,3-butadiene (1) as a system can possess potential biological activity, but compound **1** is not a good candidate for a drug. In particular, nitrodiene **1** has too few atoms in its structure, therefore, nitrodiene **1** also has insufficient molecular weight. After all, according to the PASS analysis, the (1*E*,3*E*)-1,4-dinitro-1,3-butadiene (**1**) can be active, among others, as an inhibitor of *Saccharopepsin*, *Acrocylindropepsin*, and *Polyporopepsin* as well as a treatment for pancreatic disorders.

The presented computational study shows that the future prospective for (1*E*,3*E*)-1,4-dinitro-1,3-butadiene (**1**) is its application as a building block in synthesis of carbo- and heterocyclic systems. For this purpose, the protocol of cycloaddition is one of the most significant reactions. Thanks to the protocol it is possible to obtain mostly five- and six-membered rings. Future directions for compounds thus obtained might be determined through conversion of the nitro group to other functional groups to obtain complex cyclic systems with desired structure and properties, as well as 6π-electrocyclization reactions to create bigger polycyclic systems. 

## Figures and Tables

**Table 1:** Acidic and basic agents together with yields for the condensation reaction between nitromethane (**4**) and glyoxal (**5**).

Acidic Agent	Basic Agent	Yield	Reference
SO_2_	NaOH	80.5	Novikov et al. [39]
SO_2_	NaOH	61.0	Durden et al. [35]
SO_2_	KOH	36.4	Carroll [40]
H_2_SO_4_ or H_3_PO_4_ or AcOH or (COOH)_2_	KOH	24.0	Plaut [41]

**Table 2:** Results for the choice of solvent for the extraction of 1,4-dinitrobutane-2,3-diol (**3**) from the reaction mixture (✓✓✓—soluble, ✓✓—slightly soluble, ✓—barely soluble, x—insoluble). The solubility of diol **3** was controlled by TLC, the solubility of “colored substances” was assessed by the color of the washings (it ranged from brown to pale-yellow), while the solubility of potassium acetate was tested on a pure substance.

Fraction	Solvent					
	DMFA	Nitromethane	Methanol	Acetone	Chloroform	Diethyl Ether
	(ε = 37.781)	(ε = 36.562)	(ε = 32.613)	(ε = 20.493)	(ε = 4.7113)	(ε = 4.240)
1,4-dinitrobutane-2,3-diol (**3**)	✓✓✓	✓✓✓	✓✓✓	✓✓✓	**x**	✓✓
Colored substances	✓✓✓	✓✓✓	✓✓✓	✓✓✓	✓✓✓	✓
Potassium acetate	✓✓✓	**x**	✓✓✓	✓✓✓	**x**	**x**

**Table 3:** Thermodynamic parameters of dehydro-acetylation reaction of 2,3-diacetoxy-1,4-dinitrobutane (**3a**) to 1,4-dinitro-1,3-butadiene (**1**), calculated in gas phase according to B3LYP/6-31G(d) (ΔH and ΔG are given in kcal·mol^−1^, ΔS is given in cal·mol^−1^·K^−1^).

Transformation	ΔH	ΔG	ΔS
**3a-*RR*** → **1*E*,3*E*** + 2AcOH	**33.64**	**11.98**	72.61
**3a-*RR*** → **1*E*,3*Z*** + 2AcOH	36.09	14.27	73.17
**3a-*RR*** → **1*Z*,3*Z*** + 2AcOH	37.31	15.11	74.45
**3a-*RS*** → **1*E*,3*E*** + 2AcOH	**30.31**	**7.65**	75.99
**3a-*RS*** → **1*E*,3Z** + 2AcOH	32.76	9.94	76.54
**3a-*RS*** → **1*Z*,3*Z*** + 2AcOH	33.98	10.78	77.83

**Table 4:** B3LYP/6-31G(d); the most significant ELF valence basin populations N for molecules **1** and **1′**, given an average number of electrons [e].

	1	1′
ELF Basins	N [e]	N [e]
V(C1,C2)	1.73	1.72
V’(C1,C2)	1.73	1.72
V(C2,C3)	2.23	2.20
V(C1,C2)	1.73	1.72
V’(C1,C2)	1.73	1.72

**Table 5:** B3LYP/6-31G(d) HOMO and LUMO energies as well as global reactivity indices given in electronvolts [eV].

[eV]	1	1′
HOMO energy	−8.31	−6.23
LUMO energy	−3.91	−0.61
Energy gap, ΔE	4.40	5.62
Ionization potential, l	8.31	6.23
Electron affinity, A	3.91	0.61
Electronic chemical potential, μ	−6.11	−3.42
Mulliken electronegativity, X	−4.40	−5.62
Chemical hardness, η	4.40	5.62
Chemical softness, S	0.23	0.18
Global electrophilicity, ω	4.24	1.04
Global nucleophilicity, N	0.81	2.89

**Table 6:** Drug-likeness parameters for (1*E*,3*E*)-1,4-dinitro-1,3-butadiene (**1**).

Physicochemical Properties														
Formula							C_4_H_4_N_2_O_4_							
Molecular weight MW							144.09 g/mol							
#heavy atoms							10							
#aromatic heavy atoms							0							
#rotatable bonds							3							
#H-bond acceptors							4							
#H-bond donors							0							
Molar refractivity MR							36.6							
Topological polar surface area TPSA							91.64 Å^2^							
**Lipophilicity Log P_o/w_**														
iLOGP		XLOGP		WLOGP				MLOGP		SILICOS-IT			Consensus	
−1.9		0.81		1.61				−0.78		−2.34			−0.52	
**Water Solubility Log S**														
Log S (ESOL)		Solubility		Class				Log S (Ali)		Solubility			Class	
−1.05		1.300 mg/mL		very soluble				−2.32		0.696 mg/mL			soluble	
**Pharmacokinetics**														
IG absorption	BBB permeant		CYP1A2 INH			CYP2C19 INH		CYP2C9 INH	CYP2D6 INH			CYP3A4 INH		Log K_p_ Skin permeation
High	No		No			No		No	No			No		−6.60 cm/s
**Medicinal Chemistry Friendliness**														
PAINS					Brenk						Synthetic accessibility			
0 alert					2 alerts						29.5%			

**Table 7:** Main features of the five drug-likeness rules evaluated throughout this work.

Lipinski et al. [62] (Pfizer)	Ghose et al. [63] (Amgen)	Veber et al. [64] (GSK)	Egan et al. [65] (Pharmacia)	Muegge et al. [66] (Bayer)
MW ≤ 500 Da MLOGP ≤ 4.15 #H-bond donors ≤ 5 #H-bond acceptors ≤ 10	160 Da ≤ MW ≤ 480 Da −0.4 ≤ WLOGP ≤ 5.6 40 ≤ MR ≤ 130 20 ≤ #atoms ≤ 70	#rotatable bonds ≤ 10 TPSA ≤ 140 Å^2^	WLOGP ≤ 5.88 TPSA ≤ 131.6 Å^2^	200 Da ≤ MW ≤ 600 Da −0.4 ≤ XLOGP ≤ 5.6 TPSA ≤ 150 Å^2^ #rings ≤ 7 #carbons > 4 #heteroatoms > 1 #rotatable bonds ≤ 15 #H-bond donors ≤ 5 #H-bond acceptors ≤ 10

**Table 8:** Prediction of the main antimicrobial activities of the (1E,3E)-1,4-dinitro-1,3-butadiene (**1**) using PASS. The results are expressed as a molecule’s probability to be active (Pa) or inactive (Pi).

Antimicrobial Activity	Pa	Pi
Antiviral (Picornavirus)	0.581	0.024
Antifungal	0.416	0.047
Antibacterial	0.396	0.031
Antiparasitic	0.274	0.065

## Data Availability

The data presented in this study are available on request from the corresponding author.

## Supplementary Materials

The following supporting information can be downloaded at: https://www.mdpi.com/article/10.3390/molecules29020542/s1, Experimental details: pp. S2–S4, Computational data: pp. S5, Comprehensive PASS details: pp. S6–S7.
