# Supplementary material for: (1E,3E)-1,4-Dinitro-1,3-butadiene—Synthesis, Spectral Characteristics and Computational Study Based on MEDT, ADME and PASS Simulation
Source: Molecules. 2024 Jan 22;29(2):542. doi: 10.3390/molecules29020542 (PMC10821504; doi:10.3390/molecules29020542)
Supplement: Supplementary file 1 [file molecules-29-00542-s001.zip › molecules-2815187-supplementary.pdf]

SUPPLEMENTARY MATERIALS

(1E,3E)-1,4-dinitro-1,3-butadiene – synthesis, spectral characteristic and computational study based on MEDT, ADME and PASS simulation

Mikołaj Sadowski [1], Beata Synkiewicz-Musialska [2] and Karolina Kula [1,*]

[1] Department of Organic Chemistry and Technology, Cracow University of Technology, Warszawska 24, 31-155 Cracow, Poland;

[2] Institute of Microelectronics and Photonics, Łukasiewicz Research Network, Zabłocie 39, 30-701 Kraków, Poland

* Correspondence adress: karolina.kula@pk.edu.pl (K.K)

PHYSICAL CHARACTERISTICS

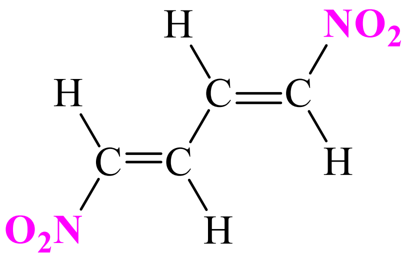

(1E,3E)-1,4-dinitro-1,3-butadiene (**1**):
UV-Vis: λ [nm] 281 (CH_3OH);
FT-IR (ATR): υ [cm^{-1}]
3106 and 3059 (~C–H stretch, alkene, medium),
1749 (>C=C< stretch, *trans* alkene, weak);
1606 (C=C< stretch, conjugated alkene, medium),
1502 (~N–O stretch, asymmetrical, nitro group, strong),
1342 (~N–O stretch, symmetrical, nitro group, strong),
985 (>C=C< bend, *trans* alkene, strong);
^{1}H NMR (400 MHz, $CDCl_3$): δ [ppm] 7.63 (d, 1H, C**H**–NO_2, J = 9.6 Hz), 7.63 (dd, 1H, =C**H**–, J_1 = 3.2 Hz, J_1 = 9.6 Hz), 7.48 (dd, 1H, =C**H**–, J_1 = 3.2 Hz, J_1 = 9.7 Hz), 7.47 (d, 1H, C**H**–NO_2, J = 9.7 Hz);
^{13}C NMR (100 MHz, $CDCl_3$): δ [ppm] 146.60 (C1 and C4); 129.50 (C2 and C3).

UV-Vis

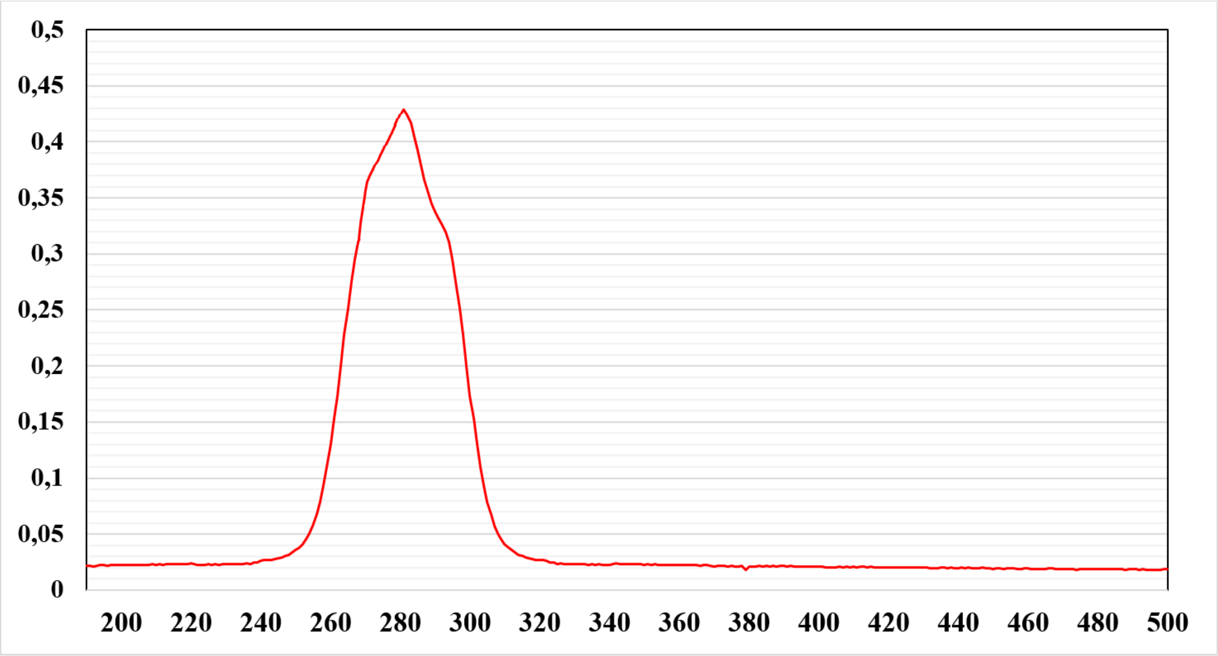

Figure S1. UV-Vis spectrum of (1E,3E)-1,4-dinitro-1,3-butadiene (**1**).

FT-IR

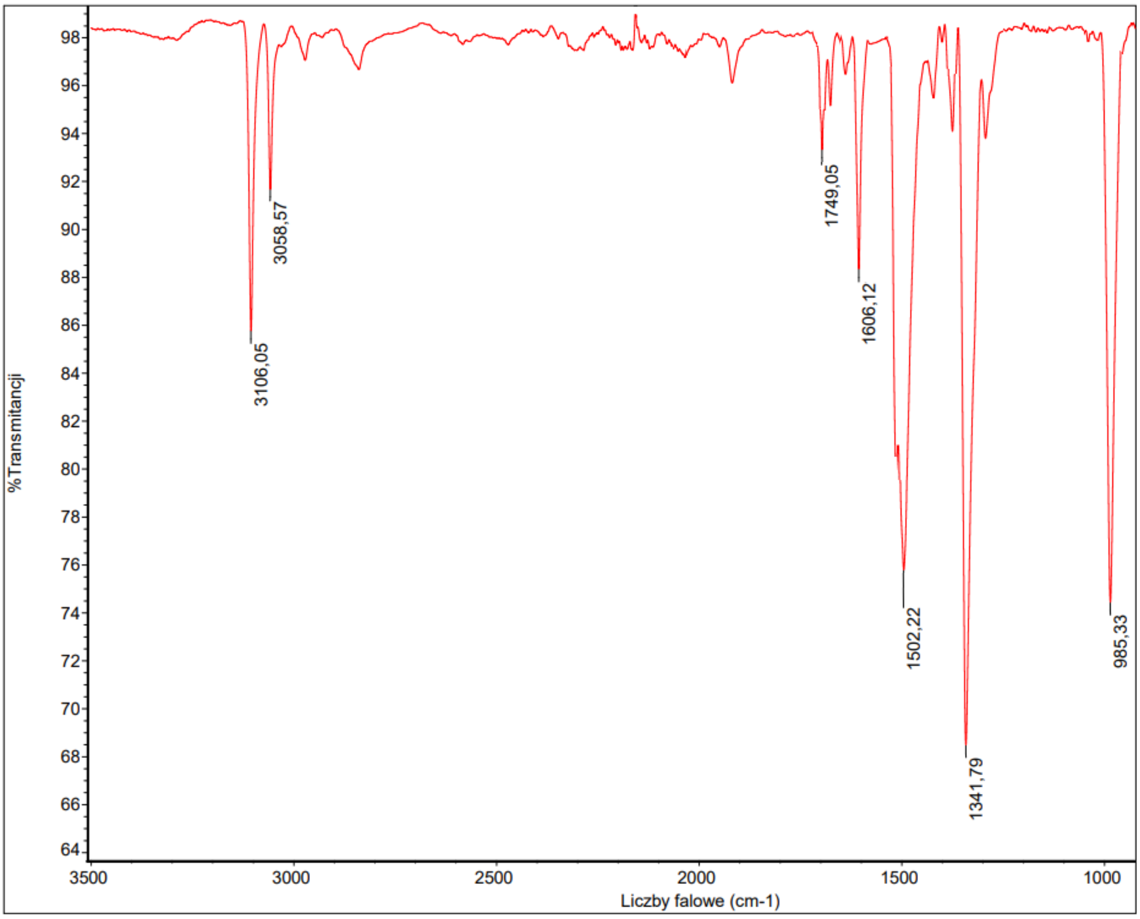

Figure S2. FT-IR spectrum of (1E,3E)-1,4-dinitro-1,3-butadiene (**1**).

^{1}H NMR

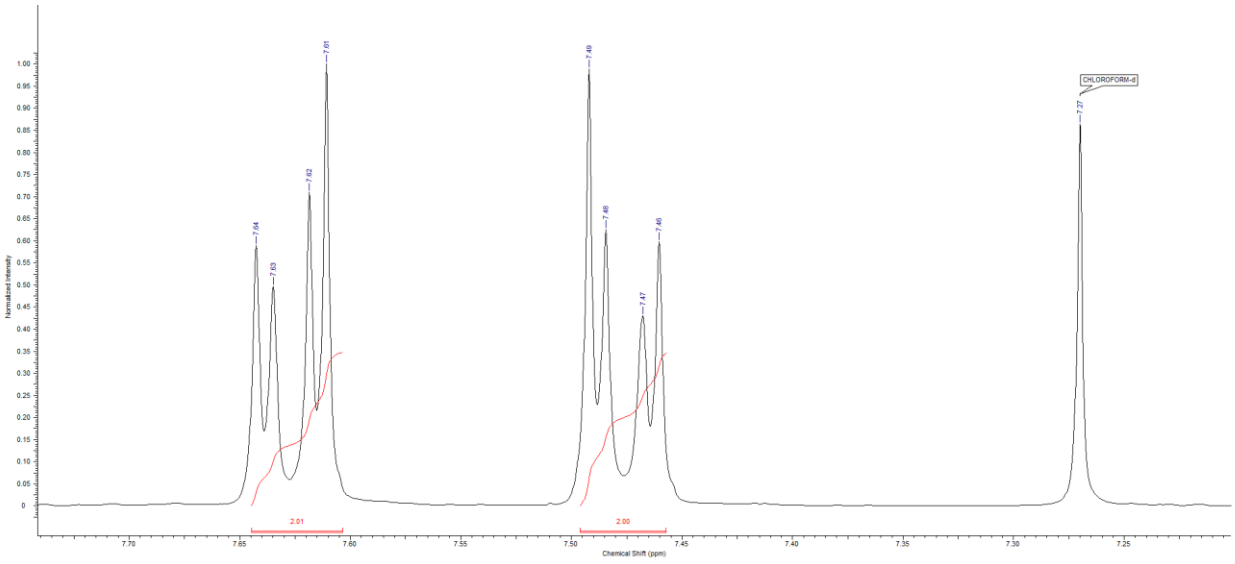

Figure S3. ^{1}H NMR spectrum of (1E,3E)-1,4-dinitro-1,3-butadiene (**1**).

^{13}C NMR

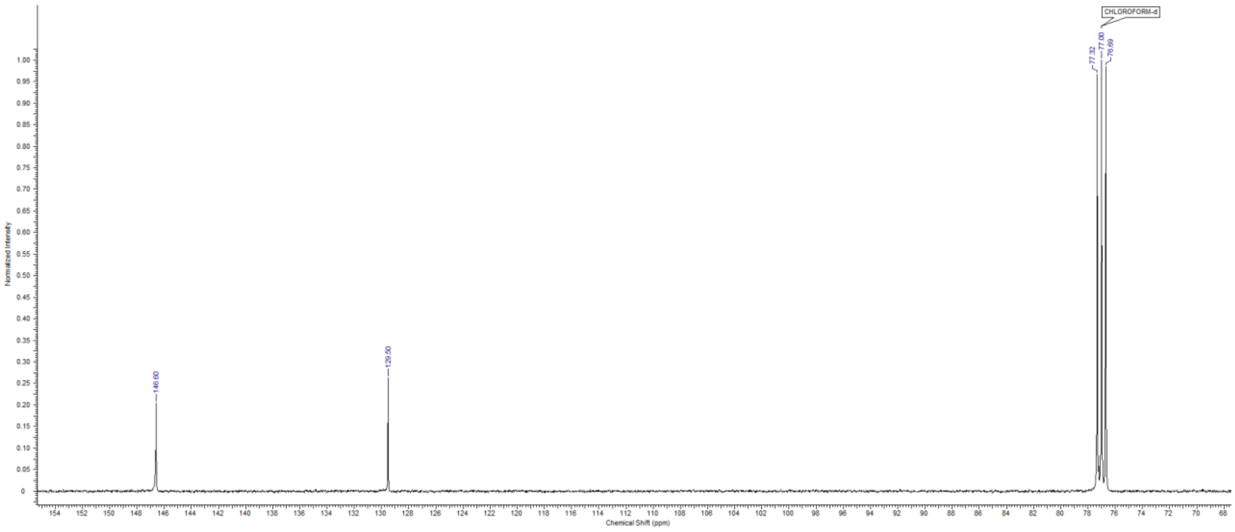

Figure S4. ^{1}H NMR spectrum of (1E,3E)-1,4-dinitro-1,3-butadiene (**1**).

HMQC

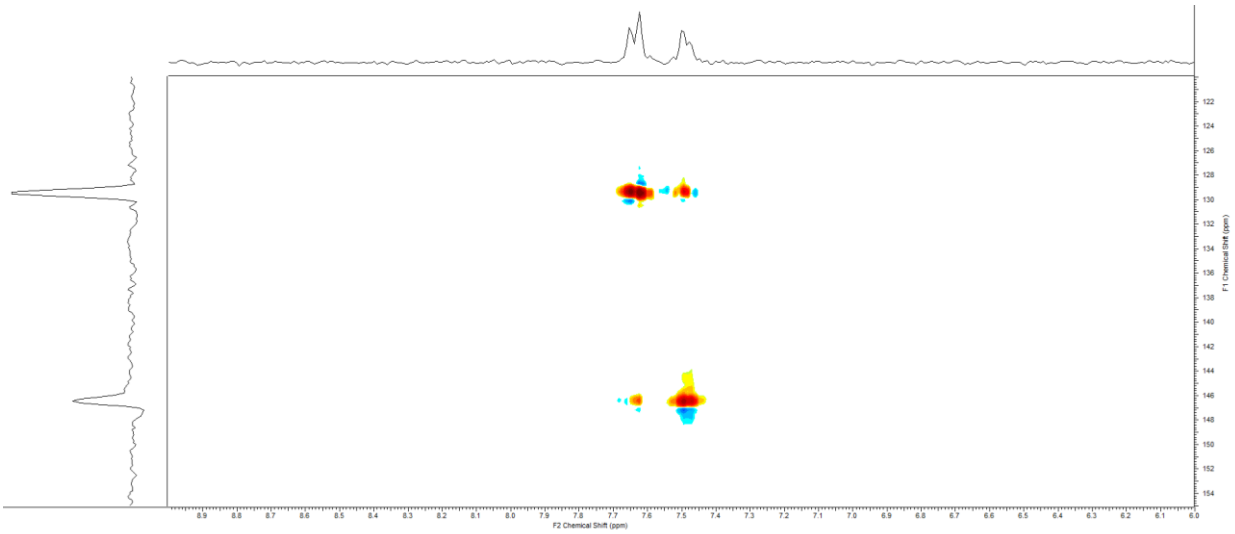

Figure S5. 2D ^{1}H-^{13}C HMQC NMR spectrum of (1E,3E)-1,4-dinitro-1,3-butadiene (**1**).

Table S1. Thermochemistry and cartesian coordinates of (1E,3E)-1,4-dinitro-1,3-butadiene (**1**) (B3LYP/6-31G(d), gas phase).

Zero-point correction =	0.091923 (Hartree/Particle)	**1**
Thermal correction to Energy =	0.101188 (Hartree/Particle)	
Thermal correction to Enthalpy =	0.102132 (Hartree/Particle)	
Thermal correction to Gibbs Free Energy =	0.055663 (Hartree/Particle)	
Sum of electronic and zero-point Energies =	-564.898200 (Hartree/Particle)	
Sum of electronic and thermal Energies =	-564.888935 (Hartree/Particle)	
Sum of electronic and thermal Enthalpies =	-564.887991 (Hartree/Particle)	
Sum of electronic and thermal Free Energies =	-564.934460 (Hartree/Particle)	

Center	Coordinates (Angstroms)		
	X	Y	Z
C	-0.67478800	0.26019800	-0.00001300
C	-1.74894700	-0.54117400	0.00007900
H	-0.83429000	1.33511200	-0.00008100
N	-3.09583400	0.01451300	0.00011100
O	-3.23204000	1.23800400	0.00005000
O	-4.01099700	-0.80958200	0.00019300
C	0.67483900	-0.26016800	-0.00001600
H	0.83434300	-1.33508500	0.00005400
C	1.74899200	0.54120400	-0.00011100
N	3.09584100	-0.01452300	-0.00012600
O	4.01101300	0.80956700	-0.00019400
O	3.23192900	-1.23803100	0.00001700
H	-1.74652700	-1.62326800	0.00015900
H	1.74660400	1.62329900	-0.00019200

Table S2. Thermochemistry and cartesian coordinates of S-trans-1,3-butadiene (**1′**) (B3LYP/6-31G(d), gas phase).

Zero-point correction =	0.085477 (Hartree/Particle)	**1′**
Thermal correction to Energy =	0.090124 (Hartree/Particle)	
Thermal correction to Enthalpy =	0.091068 (Hartree/Particle)	
Thermal correction to Gibbs Free Energy =	0.059030 (Hartree/Particle)	
Sum of electronic and zero-point Energies =	-155.906662 (Hartree/Particle)	
Sum of electronic and thermal Energies =	-155.902015 (Hartree/Particle)	
Sum of electronic and thermal Enthalpies =	-155.901071 (Hartree/Particle)	
Sum of electronic and thermal Free Energies =	-155.933110 (Hartree/Particle)	

Center	Coordinates (Angstroms)		
	X	Y	Z
C	0.60879200	-0.40063700	-0.00002500
H	0.47415500	-1.48276800	-0.00004100
C	-0.60875200	0.40049300	-0.00000700
H	-0.47387400	1.48261500	-0.00006200
C	-1.84869500	-0.10901100	0.00002500
H	-2.02270000	-1.18294800	0.00001400
C	1.84862900	0.10911900	0.00001100
H	2.02249500	1.18305500	0.00003700
H	2.72910900	-0.52625800	0.00004300
H	-2.72902500	0.52651900	-0.00001600

Table S3. Prediction of the selected activity (Pa > 0.7) of (1E,3E)-1,4-dinitro-1,3-butadiene (**1**) using PASS software. The results are expressed as probability to be active (Pa) or inactive (Pi).

	**Pa**	**Pi**
Saccharopepsin inhibitor	0.912	0.004
Acrocylindropepsin inhibitor	0.912	0.004
Chymosin inhibitor	0.912	0.004
Arachidonate-CoA ligase inhibitor	0.899	0.000
Ubiquinol-cytochrome-c reductase inhibitor	0.873	0.011
Polyporopepsin inhibitor	0.864	0.010
Aspulvinone dimethylallyltransferase inhibitor	0.861	0.017
Glucan endo-1,6-beta-glucosidase inhibitor	0.851	0.004
Bisphosphoglycerate phosphatase inhibitor	0.844	0.004
Interleukin 8 antagonist	0.836	0.003
Glycosylphosphatidylinositol phospholipase D inhibitor	0.829	0.009
Fusarinine-C ornithinesterase inhibitor	0.828	0.005
Testosterone 17beta-dehydrogenase (NADP+) inhibitor	0.827	0.019
Pancreatic disorders treatment	0.826	0.002
Arylacetonitrilase inhibitor	0.824	0.008
L-glutamate oxidase inhibitor	0.820	0.004
Phospholipid-translocating ATPase inhibitor	0.820	0.004
Sugar-phosphatase inhibitor	0.819	0.013
Feruloyl esterase inhibitor	0.815	0.010
Nicotinic alpha6beta3beta4alpha5 receptor antagonist	0.814	0.009
Phobic disorders treatment	0.811	0.030
Cutinase inhibitor	0.808	0.005
Dehydro-L-gulonate decarboxylase inhibitor	0.804	0.009
NADPH peroxidase inhibitor	0.804	0.012
Chloride peroxidase inhibitor	0.803	0.004
Pro-opiomelanocortin converting enzyme inhibitor	0.803	0.014
Carboxypeptidase Taq inhibitor	0.802	0.007
Glutamyl endopeptidase II inhibitor	0.798	0.010
Complement factor D inhibitor	0.796	0.007
Arylalkyl acylamidase inhibitor	0.795	0.004
CYP2J substrate	0.788	0.024
Poly(alpha-L-guluronate) lyase inhibitor	0.785	0.005
5-O-(4-coumaroyl)-D-quinate 3'-monooxygenase inhibitor	0.784	0.010
Phosphatidylcholine-retinol O-acyltransferase inhibitor	0.784	0.007
Taurine dehydrogenase inhibitor	0.784	0.015
Nicotinic alpha2beta2 receptor antagonist	0.780	0.013
Phthalate 4,5-dioxygenase inhibitor	0.780	0.005
Ribulose-phosphate 3-epimerase inhibitor	0.779	0.009
(R)-6-hydroxynicotine oxidase inhibitor	0.777	0.004
Bothrolysin inhibitor	0.768	0.004
Electron-transferring-flavoprotein dehydrogenase inhibitor	0.766	0.005
Chenodeoxycholoyltaurine hydrolase inhibitor	0.765	0.005
Pullulanase inhibitor	0.761	0.011
Fatty-acyl-CoA synthase inhibitor	0.760	0.007
Glutathione thiolesterase inhibitor	0.760	0.011
UDP-N-acetylglucosamine 4-epimerase inhibitor	0.755	0.010
Aldehyde dehydrogenase (pyrroloquinoline-quinone) inhibitor	0.754	0.005
All-trans-retinyl-palmitate hydrolase inhibitor	0.754	0.006
GST A substrate	0.754	0.015
Lysostaphin inhibitor	0.752	0.005
Creatininase inhibitor	0.751	0.009
Pterin deaminase inhibitor	0.747	0.007

	Pa	Pi
CYP2J2 substrate	0.746	0.023
Mucomembranous protector	0.744	0.037
N-acylmannosamine kinase inhibitor	0.743	0.005
Acylcarnitine hydrolase inhibitor	0.738	0.021
Phosphatidylserine decarboxylase inhibitor	0.738	0.008
Spermidine dehydrogenase inhibitor	0.738	0.007
Urethanase inhibitor	0.738	0.006
Allyl-alcohol dehydrogenase inhibitor	0.737	0.005
Thioredoxin inhibitor	0.737	0.007
Carnitinamidase inhibitor	0.736	0.006
Cl--transporting ATPase inhibitor	0.734	0.011
Alkylacetylglycerophosphatase inhibitor	0.733	0.016
Glutamine-phenylpyruvate transaminase inhibitor	0.733	0.008
Polyneuridine-aldehyde esterase inhibitor	0.732	0.004
tRNA-pseudouridine synthase I inhibitor	0.732	0.005
Superoxide dismutase inhibitor	0.727	0.011
Mucinaminylserine mucinaminidase inhibitor	0.726	0.009
Polyamine-transporting ATPase inhibitor	0.726	0.010
Fragilysin inhibitor	0.725	0.014
Ferredoxin-NAD+ reductase inhibitor	0.724	0.005
Naphthalene 1,2-dioxygenase inhibitor	0.724	0.005
(S)-6-hydroxynicotine oxidase inhibitor	0.722	0.005
Alkane 1-monooxygenase inhibitor	0.721	0.014
Arylsulfate sulfotransferase inhibitor	0.721	0.013
Gluconate 5-dehydrogenase inhibitor	0.721	0.009
4-Hydroxyproline epimerase inhibitor	0.718	0.006
Lysase inhibitor	0.718	0.020
NADPH-cytochrome-c2 reductase inhibitor	0.718	0.015
Pseudolysin inhibitor	0.717	0.017
Alkenylglycerophosphocholine hydrolase inhibitor	0.715	0.029
Glucan 1,4-alpha-maltotriohydrolase inhibitor	0.715	0.008
Leucolysin inhibitor	0.715	0.005
Chlordecone reductase inhibitor	0.713	0.037
IgA-specific serine endopeptidase inhibitor	0.709	0.013
Dimethylargininase inhibitor	0.702	0.014
Omptin inhibitor	0.702	0.021
